# Supplementary material for: Protein-lipid interaction at low pH induces oligomerization of the MakA cytotoxin from Vibrio cholerae
Source: eLife. 2022 Feb 8;11:e73439. doi: 10.7554/eLife.73439 (PMC8824476; doi:10.7554/eLife.73439)
Supplement: Figure 1—source data 2. [file elife-73439-fig1-data2.pdf]

Figure 1–source data 2
Original western blot figure for Figure 1E

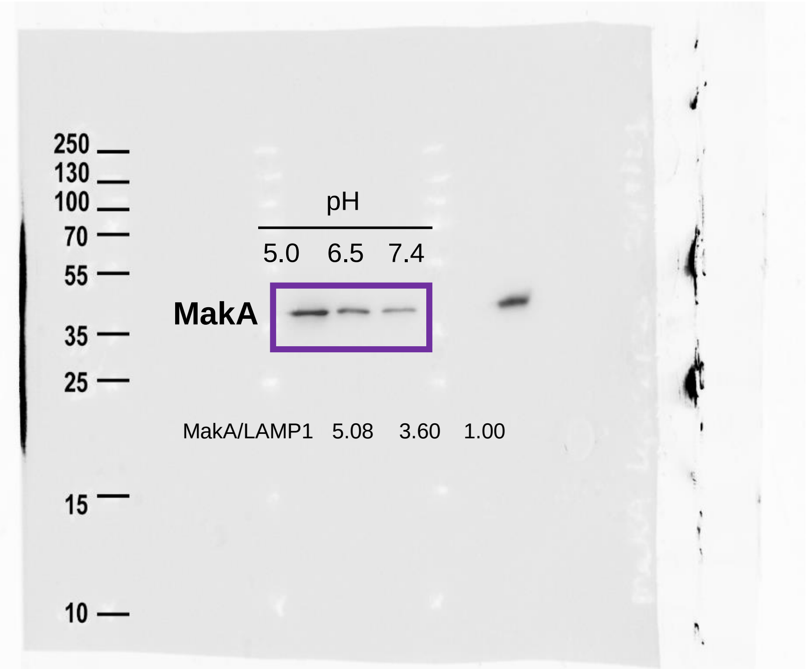

Uncropped membrane of MakA immunoblot

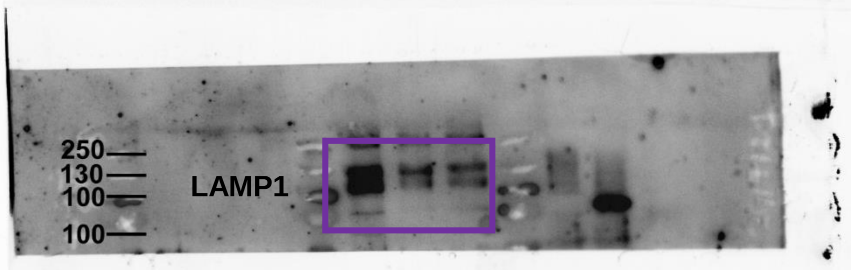

MakA immunoblot membrane was reprobed with LAMP1 antiserum
