# Supplementary figures and images for: Protein-lipid interaction at low pH induces oligomerization of the MakA cytotoxin from Vibrio cholerae
Source: eLife. 2022 Feb 8;11:e73439. doi: 10.7554/eLife.73439 (PMC8824476; doi:10.7554/eLife.73439)

**Figure 1—Figure supplement 1B—source data 1**  
**Original Western blots for MakA.**

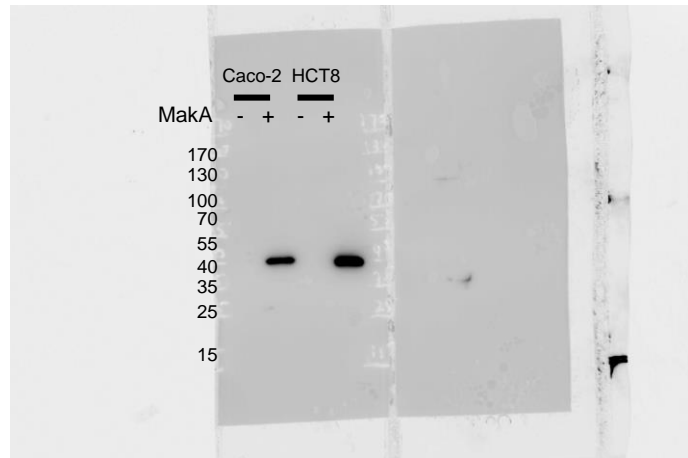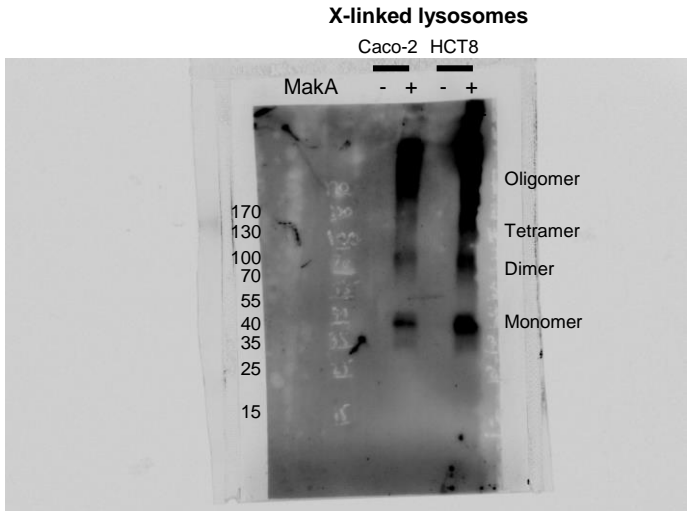

Supplement: Figure 1—figure supplement 1—source data 1. [file elife-73439-fig1-figsupp1-data1.pdf]

**Figure 2—source data 1**  
**Original western blot figure for Figure 2A**

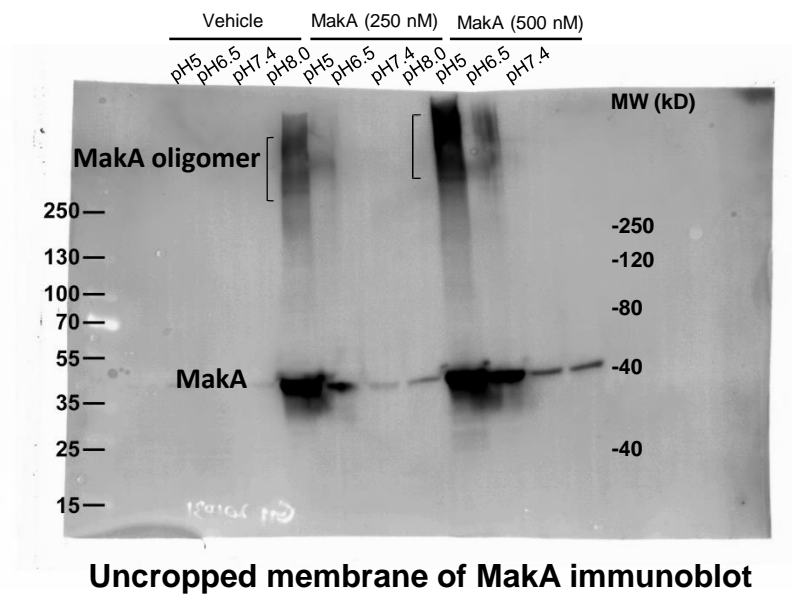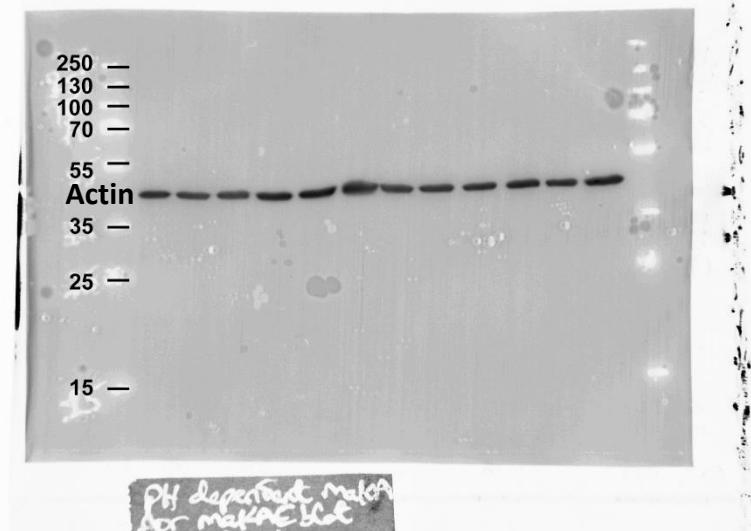

Supplement: Figure 2—source data 1. [file elife-73439-fig2-data1.pdf]

## Figure 3—figure supplement 1—source data 1

Original western blot for Figure 3—figure supplement 1A.

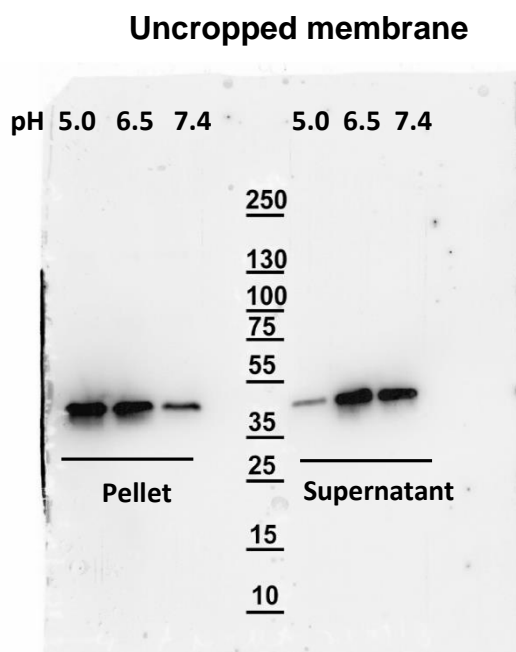

Supplement: Figure 3—figure supplement 1—source data 1. [file elife-73439-fig3-figsupp1-data1.pdf]
